# Subgroup Analysis of Overall Survival among Smoking and Non-Smoking Elderly Patients with HNSCC

**DOI:** 10.3390/cancers15061842

**Published:** 2023-03-19

**Authors:** Raphaela Graessle, Carmen Stromberger, Marcus Beck, Max Heiland, Veit M. Hofmann, Heidi Olze, Steffen Dommerich, Ulrich Gauger, Iris Piwonski, Annekatrin Coordes

**Affiliations:** 1 Department of Otorhinolaryngology, Head and Neck Surgery, Campus Virchow Klinikum and Campus Charité Mitte, Charité—Universitätsmedizin Berlin, Corporate Member of Freie Universität Berlin and Humboldt-Universität zu Berlin, 10117 Berlin, Germany; 2 Department of Radiooncology, Charité—Universitätsmedizin Berlin, Corporate Member of Freie Universität Berlin and Humboldt-Universität zu Berlin, 10117 Berlin, Germany; 3 Department of Oral and Maxillofacial Surgery, Campus Virchow Klinikum and Campus Benjamin Franklin, Charité—Universitätsmedizin Berlin, Corporate Member of Freie Universität Berlin and Humboldt-Universität zu Berlin, 10117 Berlin, Germany; 4 Department of Otorhinolaryngology, Head and Neck Surgery, Campus Benjamin Franklin, Charité—Universitätsmedizin Berlin, Corporate Member of Freie Universität Berlin and Humboldt-Universität zu Berlin, 10117 Berlin, Germany; 5 Private Statistical Consultant, 10437 Berlin, Germany; 6 Department of Pathology, Charité—Universitätsmedizin Berlin, Corporate Member of Freie Universität Berlin and Humboldt-Universität zu Berlin, 10117 Berlin, Germany; 7 Department of Otorhinolaryngology, Head and Neck Surgery, University Medical Centre Ruppin Brandenburg, Brandenburg Medical School, 16816 Neuruppin, Germany; 8 Faculty of Health Sciences Brandenburg, Joint Faculty of the University of Potsdam, Brandenburg University of Technology Cottbus-Senftenberg and Brandenburg Medical School, 14476 Potsdam, Germany

**Keywords:** head and neck squamous cell carcinoma, elderly patients, smoking, survival

## Abstract

**Simple Summary:**

The aim of this study was to deliver more insight into factors influencing survival of elderly patients with HNSCC. Although elderly patients have become increasingly important from a demographic point of view, studies dedicated to them are rare. We were able to confirm in a large study cohort that non-smokers with HNSCC have a significantly higher chance of surviving HNSCC than smokers. We showed that elderly non-smokers are also affected by HNSCC; however, both their overall survival and their disease-free survival are increased compared to smokers. Important predictors of survival, both in smokers and in non-smokers, were, among others, alcohol abuse, health status (Karnofsky performance status), biological age (Charlson comorbidity index), site of primary tumour, UICC stage and treatment received.

**Abstract:**

Smoking is a leading cause of head and neck squamous cell carcinoma (HNSCC). However, non-smokers are also affected by HNSCC, and the prognostic factors applicable to older non-smokers with HNSCC are largely unknown. The aim of this study was to determine predictors of overall survival (OS) in patients both with and without a smoking history aged 70 and over at initial diagnosis. Retrospective data of patients aged ≥70 (initial diagnoses 2004–2018) were examined. Evaluated predictors included tumour stage, biological age, health and therapy. A total of 688 patients (520 smokers, 168 non-smokers) were included with a median age of 74. The 5-year OS was 39.6%. Non-smokers had significantly improved OS compared to smokers (52.0% versus 36.0%, *p* < 0.001). Disease-free survival (DFS) differed significantly between both groups (hazard ratio = 1.3; 95%CI 1.04–1.626). TNM stage and the recommended therapies (curative versus palliative) were comparable. The proportion of p16-positive oropharyngeal carcinomas was significantly higher in non-smokers (76.7% versus 43.8%, *p* < 0.001). Smokers were significantly more likely to be men (*p* < 0.001), drinkers (*p* < 0.001), and have poorer health status (Karnofsky performance status, KPS, *p* = 0.023). They were also more likely to have additional tumours (*p* = 0.012) and lower treatment adherence (*p* = 0.038). Important predictors of OS identified in both groups, were, among others, alcohol abuse, KPS, Charlson comorbidity index, site of primary tumour, UICC stage and treatment received. Elderly non-smokers are also affected by HNSCC, however, both OS and DFS are increased compared to smokers.

## 1. Introduction

Carcinomas in the head and neck region, representing 3.7% of annual new cancer cases in men, are the seventh most common tumour in Germany (together with pancreatic carcinoma). In German women, they account for 1.9% of new cancer diagnoses, equal to cervical cancer. The majority (84%) of these carcinomas are squamous cell carcinomas (HNSCC). The current median age of onset is 64 years for men and 2 years later for women [1]. However, with growing life expectancy and the increasing proportion of older patients, the median onset age is also likely to increase [2,3]. There are few studies that focus on elderly patients, who typically represent a small, easily overlooked portion of a much larger patient cohort [4]. This may be because the typical head and neck squamous cell carcinoma (HNSCC) patient is a male middle-aged smoker, and frequently a drinker too. This is the case because any type of tobacco and alcohol use is an important risk factor for developing HNSCC. Studies have shown that these two factors even have a mutually reinforcing effect [1,5,6,7,8]. Smokers with HNSCC have been shown to have certain characteristics: Their smoking status is associated with poorer overall survival (OS) and poorer disease-free survival (DFS) [9,10,11,12,13]. There is even a certain positive effect on survival if smoking cessation occurs only after HNSCC is diagnosed [14]. The risk of human papillomavirus (HPV) infection, particularly HPV-16, is now also an issue of increasing focus [15]. HPV has recently become significantly more frequent in patients with HNSCC [16] and is responsible for oropharyngeal carcinoma in young non-smokers [17,18].

With the decline in smoking prevalence, a new patient group without the traditional risk factors is growing [9,11,19,20,21]. This group (i.e., non-smoking and non-drinking patients) has a higher median age and is more commonly female than the patient group with traditional risk factors [9,22]. However, as most studies that investigated the association between smoking habits and HNSCC did not focus specifically on elderly patients, it is uncertain to what extent these observations apply to the subgroup of elderly patients, which still includes a significant number of current and former smokers. In Germany, 31% of people between 70 and 75 years are former smokers and 12% of this cohort are active smokers. Only 5% of Germans older than 75 years smoke but in 27% of cases they have smoked regularly at some point in their lives [23]. Although the proportion of current smokers decreases with age, cumulative lifetime tobacco exposure also influences OS [10,23].

It is therefore questionable whether the same prognostic factors for OS apply to non-smokers in an older patient cohort as in the younger patient cohort that is usually analysed. For example, there are findings suggesting that older, non-smoking, non-drinking female patients represent a particular subgroup in oral squamous cell carcinomas with worse DFS [9,22]. The need for a more subgroup-specific investigation has therefore become increasingly clear. Therefore, this study primarily aims to find OS predictors in smokers and non-smokers older than 70 years at the time of initial diagnosis. In this context, it also compares OS and DFS in both groups. Secondarily, we investigate predictors of a negative smoking status in HNSCC patients.

## 2. Materials and Methods

### 2.1. Inclusion Criteria and Data Acquisition

In this retrospective analysis, patients who were ≥70 and diagnosed with an HNSCC between 2004 and 2018 at the Charité, Universitätsmedizin Berlin were included. All HNSCC were confirmed histologically. Squamous cell carcinomas located in the oro-/naso-/hypopharynx, the larynx, the oral cavity and the nasal/paranasal sinuses were included. During patients’ treatment and follow-up examinations, all diagnostic results and treatment decisions were documented in their electronic record. All clinicopathological data for this investigation could therefore be taken retrospectively from these medical records. In this study, the baseline requirement for inclusion was that tumour stage and smoking status were available for each patient. This requirement along with the aforementioned inclusion criteria lead to a study population of 688 patients in total. The study was approved by the local ethics committee (EA1/256/20).

### 2.2. Diagnostic and Treatment Procedure

International standard procedures were used for diagnostic evaluation. After a detailed medical history with special attention to HNSCC risk factors was obtained, a clinical examination of the head and neck region followed. This examination included an assessment of the laryngeal and pharyngeal region using an endoscope, followed by radiological imaging such as a computed tomography (CT) scan or magnetic resonance imaging (MRI) of the head and neck region. A CT scan of the thorax and abdomen was also performed to exclude distal metastases. A pan-endoscopy with a tissue biopsy was performed for histological confirmation. These diagnostic steps also determined the expansion of the tumour and the presence of any secondary malignancies. Since the 8th edition of the Union for International Cancer Control (UICC) TNM classification was released in 2017, all biopsies of oropharyngeal squamous cell carcinomas were routinely investigated regarding the expression of p16 as a surrogate marker for HPV association [24,25]. Tissue samples collected between 2004 and 2017 had already been examined for p16 in some cases; otherwise, attempts were made to examine the corresponding archived tissue samples using immunohistochemistry. Subsequent staining did not work in all cases. For this reason, oropharyngeal carcinomas in which no tissue was available for subsequent staining were classified as p16 negative for tumour staging. They were excluded from all other p16 specific analyses. For this study, all tumour classifications were evaluated according to the 8th edition of the UICC TNM classification in order to ensure comparability. Tumours initially classified according to the 7th edition were adapted to the new edition retrospectively.

Patient therapies were discussed by a multidisciplinary tumour board consisting of head and neck surgeons, radiologists, medical and radiation oncologists and pathologists. Therapy decisions were made in cooperation with the patient following the shared decision-making method. Patients who followed the recommendations of the tumour board were rated as adherent. All others were classified as non-adherent. Tumour board decisions were generally based on the internationally recognized National Comprehensive Cancer Network’s (NCCN) guidelines, but also considered patient-specific factors that influence the therapy, such as the general condition or comorbidities that may limit therapy options [26]. For this study, the medical condition and the comorbidities prior to the tumour therapy were extracted from the patients’ records. Patients’ medical conditions were evaluated using the Karnofsky performance status (KPS) [27]. The KPS was either documented before the therapy or could easily be deduced from the anamnestic information contained in the records. Comorbidities were scored retrospectively using the Charlson comorbidity index (CCI) [28]. As recommended by Charlson et al. [28], we used both age and comorbidity as predictors of death, as our follow-up periods were generally greater than 5 years. Therefore, all patients aged 70 years scored 3 points on the CCI regardless of their comorbidities. Another point was added for every additional decade. It was attempted to excise resectable tumours in sano. Adjuvant radio(chemo)therapy (adj. RT/RCT) was recommended when patients suffered from advanced tumour disease (UICC > II), tumour tissue remained in situ, close surgical margin status was found, or extracapsular lymph node spread was confirmed histologically. Whenever excision was not possible, definitive radio(chemo)therapy (def. RT/RCT) remained a curative option. Palliative treatment options included palliative radio(chemo)therapy (pall. RT/RCT), systemic therapy (ST) including chemotherapy or immunotherapy and best supportive care (BSC). Follow-up consisted of regular examinations, pan-endoscopies and imaging (CT, MRI and ultrasound). During the first year after therapy, patients were encouraged to attend check-ups every 1–3 months. Examination intervals were extended gradually thereafter.

### 2.3. Statistical Analysis

All statistical calculations were performed using SPSS Statistics version 28.0.1.0 for macOS (IBM Corp., Armonk, NY, USA) and R version 4.2.2. All data were reproduced in accordance with the SAMPLE Guidelines [29]. As an exploratory data analysis was conducted in this study, all *p*-values were presented without adjustment for multiple testing. For all analyses, *p*-values < 0.05 were considered statistically significant.

In the study population, the following patient characteristics were included: sex (male vs. female), age at initial diagnosis of HNSCC, adherence to medical treatment recommendations (adherent vs. non-adherent), smoking status (current/former smokers vs. non-smokers), pack years (PY), alcohol abuse (no ethanol consumption vs. ethanol consumption), additional cancer diagnoses (other cancers vs. none), number of additional cancer diagnosis (0 vs. 1 vs. ≥2), CCI (≤5 vs. ≥6), KPS (≤70% vs. ≥80%), death due to cancer (survived vs. cancer-associated vs. non-cancer-associated), tumour site (oropharynx, oral cavity, larynx, hypopharynx, nasal/paranasal sinus, nasopharynx), p16 expression (in oropharyngeal carcinomas only; positive vs. negative), histological grading (G1 vs. G2 vs. G3), T classification (T1–2 vs. T3–4), N classification (negative vs. positive), M classification (negative vs. positive), UICC stage (I–II vs. III–IV), received treatment (BSC vs. pall. RT/RCT vs. surgery vs. surgery + adj. RT/RCT vs. def. RT/RCT vs. ST), recommended treatment (BSC vs. pall. RT/RCT vs. surgery vs. surgery + adj. RT/RCT vs. def. RT/RCT vs. ST), intention of therapy (curative vs. palliative vs. curative, discontinued) and recurrence (positive vs. negative). As can be seen from the enumeration, some variables were categorised for clarity. The frequency distribution of the patient characteristics was computed for the entire study population as well as for the two subgroups (i.e., the former/current smokers and the non-smokers). All current or former smokers were classified in the ‘smokers’ subgroup. Patients who never smoked were categorised in the ‘non-smokers’ subgroup. To evaluate the differences in constitution between the two subgroups, a chi-square test was performed for all categorial variables. All metric variables (age at initial diagnosis of HNSCC, PY) were tested for normal distribution. Since all metric variables turned out to be non-normally distributed, the Mann–Whitney U-test was used to evaluate the differences among them.

To evaluate the OS and the DFS of the study population, the Kaplan–Meier method was used for the univariate analysis. OS was defined as the time between initial diagnosis of HNSCC and death from any cause or last follow-up. DFS was calculated from the time between the initial diagnosis of HNSCC and the first recurrence of the primary cancer or last follow-up. DFS was a maximum of 5 years. After this period, the carcinomas were recorded as secondary carcinomas rather than recurrences. To assess variables with significant influence on OS, the log-rank test was applied. These analyses were performed once for the entire study population, and once for the two subgroups separately. Thus, the variables influencing OS generally and the differences in influence between the two subgroups could be determined. For the OS analysis, sex, age at initial diagnosis of HNSCC, adherence to treatment recommendation, alcohol abuse, additional cancer diagnoses, CCI, KPS, tumour site, p16 expression (in oropharyngeal carcinomas only), histological grading, tumour stage and T, N and M classification, received and recommended therapy, intention of therapy and recurrence were considered. The Cox proportional hazards model was used for the multivariate analysis of OS. In this analysis, age at initial diagnosis (≤75 vs. ≥76), smoking status (non-smokers, current/former smokers), CCI (≤5 vs. ≥6), KPS (≤70% vs. ≥80%) and UICC stage (I–II vs. III–IV) were considered.

DFS was computed for the entire patient cohort using the Kaplan–Meier method. In addition, a recurrent event analysis was conducted using the Andersen–Gill counting process to further assess the impact of smoking on DFS, the relevant events being death or recurrence.

## 3. Results

### 3.1. Patient Characteristics

This study included 688 patients aged ≥70. The median age was 74 (range 26, from 70 to 96) years. A summary of all clinicopathological characteristics of the study population is shown in Table 1.

The majority (71.7%) of the patients were male. A total of 37.0% of the study population consumed large quantities of alcohol. Most patients had no history of cancer before the diagnosis of the HNSCC and had a good biological age with less than 5 points on the CCI (64.5%). A total of 315 (46.0%) patients reached ≥80% on the KPS. The largest percentage of patients suffered from carcinoma located in the oral cavity (34.9%, *n* = 240), followed by oropharynx (29.4%, *n* = 202), larynx (22.4%, *n* = 154), hypopharynx (9.0%, *n* = 62), nasal/paranasal sinuses (3.5%, *n* = 24) and nasopharynx (0.9%, *n* = 6). Half of all patients with oropharyngeal carcinomas had p16 positive tumours (51.1%, *n* = 69). Most carcinomas were detected at a locally advanced tumour stage (UICC stage III–IV: *n* = 416, 60.5%). In 4.4% (*n* = 30) of cases, the HNSCC had already formed distant metastases at the time of initial diagnosis. In 528 (77.1%) patients, the therapy intention was curative. The majority of the study population (86.1%, *n* = 551) was adherent to the recommended therapy. A total of 46 (6.7%) patients discontinued the therapy for a range of reasons. The following therapies were received with decreasing frequency: surgery (38.1%, *n* = 258), def. RT/RCT (31.3%, *n* = 212), surgery + adj. RT/RCT (14.9%, *n* = 101), pall. RT/RCT (6.8%, *n* = 46), BSC (7.2%, *n* = 49) and ST (1.6%, *n* = 11). In 152 (22.1%) patients, a recurrence of the carcinoma occurred.

### 3.2. OS, DFS and Predictors of Survival

**Figure 1:**
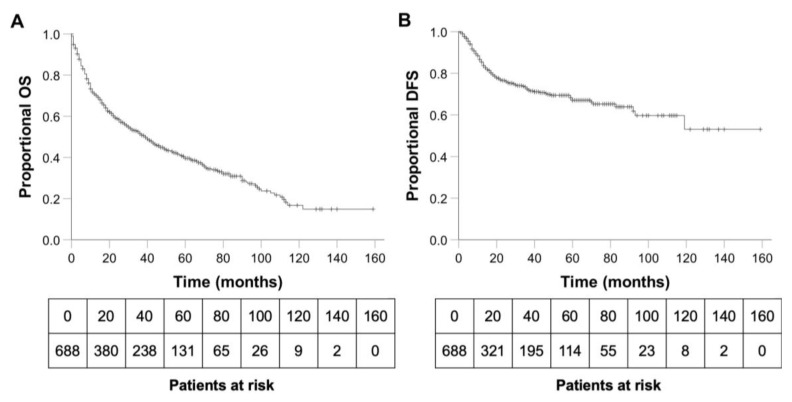
(**A**). Overall survival of the 688 patients ≥ 70 years with HNSCC. (**B**). Disease-free survival of the 688 patients ≥ 70 years with HNSCC. OS, overall survival; DFS, disease-free survival.

The mean survival of the study population was 58 months (95%CI 52.93–64.00). The 1-, 3- and 5-year OS rates were 70.9, 51.6 and 39.6%, respectively (Figure 1A). A total of 394 (57.3%) patients died during the follow-up period. A total of 256 (65.0%) of those deaths were clearly associated with head and neck tumour progression. In 70 cases, the cause of death remained unknown. In 69 cases, death was non-cancer related. Deaths of patients with (locally) advanced disease (UICC III–IV) were significantly more often associated with HNSCC compared to patients with less advanced disease, see Table 2 (*p* < 0.001). Mean DFS was 105 months (95%CI 96.24–113.87). The 1-, 3- and 5-year DFS rates were 85.4, 72.5 and 67.0%, respectively (Figure 1B).

In the univariate analysis considering all included patients, OS was influenced significantly by patients’ alcohol consumption (*p* < 0.001), biological age (CCI *p* < 0.001), general health status (KPS *p* < 0.001), location of the HNSCC (*p* < 0.001), p16 expression in oropharyngeal carcinomas (*p* = 0.022), tumour classifications (T-, N- and M-classification with *p* < 0.001 for all), UICC stage (*p* < 0.001), adherence of patients to their medically recommended therapy (*p* < 0.001), recommended and received treatment (*p* < 0.001), implementation of therapy (*p* < 0.001) and treatment intention (*p* < 0.001). Another factor affecting long-term survival was smoking status (*p* = 0.001).

### 3.3. Predictors of a Negative Smoking Status in HNSCC Patients

According to their smoking habits, patients were separated into two groups. The group of non-smokers included 168 (24.4%) patients who had never smoked during their lifetimes (0 PY). The group of smokers included 520 (75.6%) patients who were current or former smokers with a median of 49 PY (range 197, from 3 to 200).

Significantly more males were found in the smoking group compared to the non-smoking group (75.0% vs. 61.3%, *p* < 0.001). The median chronological age at initial diagnosis of HNSCC was significantly higher in the non-smoker group (74 vs. 76 years, *p* < 0.001). Affected smokers consumed alcohol (44.7%, *n* = 169) significantly more often than non-smokers (13.6%, *n* = 17, *p* < 0.001). HNSCC patients in the smoking group had a history of cancer significantly more often than patients in the non-smoking group (38.1% vs. 27.4%, *p* = 0.012). This is also reflected in the number of carcinomas before the HNSCC diagnosis (8.4% vs. 6.5% suffered from ≥2 carcinomas before the HNSCC diagnosis, *p* = 0.041). Non-smokers had a better general health condition than smokers (*p* = 0.023). A KPS ≥ 80% was reached by 53.6% (*n* = 90) of non-smoking patients, whereas 43.5% (*n* = 225) of current or former smokers achieved that score. In the non-smoking group, significantly fewer patients died of cancer than in the control group (32.1% vs. 44.6%, *p* < 0.001). Here, significantly more patients had p16 positive oropharyngeal carcinomas than in the smoking group (76.7% vs. 43.8%, *p* < 0.001). Further significant differences were found regarding the recommended treatment (*p* = 0.002) and the received treatment (*p* = 0.046). In addition, non-smoking patients adhered significantly more often to medical treatment recommendations than their smoking counterparts (91.1% vs. 84.5%, *p* = 0.038). No differences were found in the distribution of tumour site, the UICC stage (T-, N- and M-classification), the histological grading, the intention of the treatment and the recurrence.

### 3.4. Differences between Smokers and Non-Smokers Concerning OS and DFS

**Figure 2:**
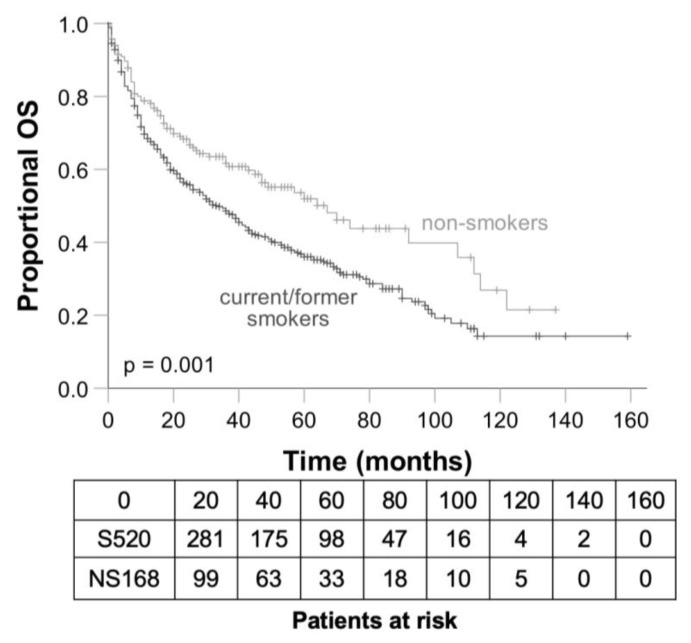
Overall survival depending on the smoking status. OS, overall survival; S, current/former smokers; NS, non-smokers.

The mean OS of smokers was 54 months (95%CI 48.38–60.58) with 1-, 3- and 5-year OS rates of 68.4, 48.6 and 36.0%, respectively. The mean OS of the non-smoking group was 69 months (95%CI 59.23–79.47) and differed significantly from the smoking group (*p* = 0.001) in which the 1-, 3- ad 5-year OS rates were 78.8, 61.7, 52.0%, respectively (Figure 2). There was also a significant influence of the smoking status on DFS (hazard ratio = 1.3; 95%CI 1.04–1.626).

Sex, additional cancer diagnosis, histological grading and tumour recurrence did not significantly influence OS overall, nor did they affect OS in the smoking and non-smoking subgroups. The p16 expression in oropharyngeal carcinoma did not significantly influence OS at the subgroup level. However, in non-smoking patients, 76.7% (23 out of 30) were p16positive. In the multivariate analysis, an independent impact on OS of age at initial diagnosis (categorised in ≤75 and ≥75), smoking status, CCI, KPS and UICC tumour stage was established. In the subgroup of smokers, the results were identical compared to the general study population. In non-smoking patients, which included the lower number of patients, only the KPS and the tumour stage achieved a significant level. All analysed factors for OS are summarised in Table 3 and Table 4.

## 4. Discussion

The aim of this study was to find OS predictors in smokers and non-smokers older than 70 at the time of initial diagnosis. A total of 688 patients (520 smokers and 168 non-smokers) over 70 years of age at initial diagnosis were analysed. The 5-year OS was 39.6%. Factors that influenced OS in non-smokers included alcohol abuse, adherence to treatment recommendations, good health, T- and M-classification and UICC stage, which was comparable to the smoking subgroup. These results are similar to those in the literature [30,31,32,33,34,35].

The term ‘non-smoker’ is not clearly defined in the literature. In this study, patients were considered non-smokers if they did not use tobacco regularly, either in the past or present. This approach was also chosen in several previous publications [10,11,12,22,36]. However, in some previous research dealing with tobacco abuse and carcinogenesis the definition of non-smoking is not so clear [21,37]. In this context, it should be considered that a patient who does not smoke at the time of cancer diagnosis may nevertheless have accumulated a significant number of PY through previous smoking and should therefore not be counted as a non-smoker. Unfortunately, due to the retrospective character of this study, we were unable to consider the effect of second-hand smoking in elderly patients. Since smoking was more socially accepted in the elder generation and environmental exposure might have been higher, this could play a major role, especially in earlier studies of older patients. Idris et al. [38] studied second-hand smoking for all patients over 18 years of age and found it to be a predictor of recurrence and lower OS. Whether this is still an issue in the subgroup of elderly patients could not be clarified in this study and provides opportunities for further research.

In the literature we found studies dealing with tobacco abuse in elderly patients [9,21,22]. However, all of these publications focussed on oral squamous cell carcinoma, which is only one of the subsites our study considers. Restricting the study to this subset of HNSCC might bring homogeneity to the study population but neglects all other HNSCC (oropharyngeal and laryngeal carcinomas) whose mortality is also influenced by smoking [33]. Moreover, two of the aforementioned studies [9,22] differentiate between non-smoking, non-drinking elderly and smoking or drinking elderly, thereby offering different classifications than our study. In those studies, the group of patients without traditional risk factors is compared to the group with traditional risk factors. This study design makes it impossible to draw conclusions about the effects of smoking as an isolated factor.

During our review, we found different age thresholds for older patients. Following thresholds used in other studies, we settled on a threshold of 70 as the lowest limit for patient enrolment [21,39,40,41]. In addition to increased age (usually ≥70 years), geriatric patients also have a certain multimorbidity and frailty [42,43]. The present study took this aspect into consideration and, unlike previous studies that addressed tobacco use in older HNSCC patients, included CCI and KPS [9,21,22]. In accordance with the literature, advanced comorbidity was shown to be associated with lower OS [30,44,45,46]. In this study, both KPS and CCI were associated with OS, but only KPS showed a significant difference between smokers and non-smokers. Since smoking is associated with several diseases, which are taken into account by the CCI with a score from 1 to 6 points [28,47,48,49], it is surprising that no difference in distribution of the CCI among both groups could be found. A possible reason for this result might be that we used an age-adjusted version of the CCI. Since there was a significant difference in age distribution, this may have biased our results regarding the CCI. Non-smokers may have had fewer comorbidities but were also older than smokers, which could result in a similar CCI score.

It remains controversial whether p16 expression in oropharyngeal cancer is associated with higher OS. Wendt et al. [50] and Marklund et al. [51] discovered an improved OS in patients with tonsillar and base-of-tongue carcinomas, whereas they did not find any significant survival advantage in other subsites of the oropharynx. Our findings support the positive impact of p16 expression on OS of elderly patients. However, in non-smoking patients the impact of p16 was not significant. This finding could be explained by the high proportion of p16-positive patients among non-smokers in our study population, which amounted to almost 80%. This could influence the results, as the subgroups of smokers and non-smokers differed significantly in terms of p16 expression [52].

Smokers are more likely to be non-adherent to recommended treatment strategies than non-smokers [30], as was corroborated by this study. However, the smoking and non-smoking patient groups did not only differ in adherence and received treatment but also in treatment recommendations. Dronkers et al. [53] examined the factors that led physicians to deviate from guidelines. They found that age and severe comorbidities were among the influencing factors. We therefore assume that the lower KPS and thus the poorer health status in smokers led to different decisions by the tumour board. This might also confound the OS differences between smokers and non-smokers since non-guideline treatment is associated with a lower OS [53].

Limitations of the study include the retrospective study design. Therefore, we had limited ability to include information such as second-hand smoking or the social status of a patient. The two compared groups differed in size; non-smokers and smokers differed significantly in some characteristics, such as sex, chronological age and general health status.

In elderly non-smoking patients with HNSCC, cancerogenesis may not stem from tobacco exposure causing field cancerization. Instead, it may come from age-associated genetic alterations, including HRAS and CASP8. In contrast to HPV-associated carcinomas, there may be fewer immune cells in the tumour microenvironment. Further investigations are necessary here [54].

## 5. Conclusions

This study gives a broad overview of clinicopathological data of elderly patients with HNSCC, treated at a large head and neck tumour centre in Germany. Non-smoking patients with HNSCC achieved significantly better 5-year OS and DFS. Factors that influenced OS in both groups included patients’ alcohol consumption, biological age (CCI), general health status (KPS), p16 association in oropharyngeal cancer, tumour stage, patient adherence to physician-recommended therapy, treatment delivery and intention of treatment. Thus, these main predictors for OS can also be applied to elderly HNSCC patients with and without smoking history. Verification of these results by prospective studies is still needed.

## Figures and Tables

**Table 1:** Patient and tumour characteristics of the study population according to their smoking status.

Variable	Total	Current/Former Smokers	Non-Smokers	*p* Value
	*n* = 688	*n* = 520	*n* = 168	
Sex—no. (%)				<0.001
Male	493 (71.7)	390 (75.0)	103 (61.3)	
Female	195 (28.3)	130 (25.0)	65 (38.7)	
Age at initial diagnosis of HNSCC, years				<0.001
Median (range)	74 (26)	74 (26)	76 (22)	
Pack years				<0.001
Median (range)	49 (197)	49 (197)	0 (0)	
Alcohol abuse—no. (%)				<0.001
No ethanol consumption	317 (63.0)	209 (55.3)	108 (86.4)	
Ethanol consumption	186 (37.0)	169 (44.7)	17 (13.6)	
Additional cancer diagnoses—no. (%)				0.012
Other cancers	244 (35.5)	198 (38.1)	46 (27.4)	
None	444 (64.5)	322 (61.9)	122 (72.6)	
Number of additional cancer diagnoses—no. (%)				0.041
0	444 (64.5)	322 (61.9)	122 (72.6)	
1	189 (27.5)	154 (29.6)	35 (20.8)	
≥2	55 (8.0)	44 (8.5)	11 (6.5)	
Charlson comorbidity index—no. (%)				0.301
≤5	444 (64.5)	330 (63.5)	114 (67.9)	
≥6	244 (35.5)	190 (36.5)	54 (32.1)	
Karnofsky performance status—no. (%)				0.023
≤70%	370 (54.0)	292 (56.5)	78 (46.4)	
≥80%	315 (46.0)	225 (43.5)	90 (53.6)	
Death due to cancer—no. (%)				<0.001
Survived	293 (47.4)	199 (43.1)	94 (60.3)	
Non-cancer-associated	69 (11.2)	57 (12.3)	12 (7.7)	
Cancer-associated	256 (41.4)	206 (44.6)	50 (32.1)	
Recurrence—no. (%)				0.406
Positive	152 (22.1)	111 (21.3)	41 (24.4)	
HNSCC characteristics				
Tumour site—no. (%)				0.066 ^1^
Oropharynx	202 (29.4)	155 (29.8)	47 (28.0)	
Oral cavity	240 (34.9)	173 (33.3)	67 (39.9)	
Larynx	154 (22.4)	116 (22.3)	38 (22.6)	
Hypopharynx	62 (9.0)	56 (10.8)	6 (3.6)	
Nasal/paranasal sinuses	24 (3.5)	16 (3.1)	8 (4.8)	
Nasopharynx	6 (0.9)	4 (0.8)	2 (1.2)	
P16 in oropharynx carcinoma—no. (%)				0.001
Positive	69 (51.1)	46 (43.8)	23 (76.7)	
Grading—no. (%)				0.387
G1	59 (9.4)	42 (8.8)	17 (11.1)	
G2	412 (65.3)	319 (66.7)	93 (60.8)	
G3	160 (25.4)	117 (24.5)	43 (28.1)	
T classification—no. (%)				0.121
T1–2	341 (49.6)	249 (47.9)	92 (54.8)	
T3–4	347 (50.4)	271 (52.1)	76 (45.2)	
N classification—no. (%)				0.121
Positive	347 (50.4)	271 (52.1)	76 (45.2)	
M classification—no. (%)				0.887
Positive	30 (4.4)	23 (4.4)	7 (4.2)	
UICC stage (8th edition)—no. (%)				0.119
0–II	272 (39.5)	197 (37.9)	75 (44.6)	
III–IV	416 (60.5)	323 (62.1)	93 (55.4)	
Intention of therapy—no. (%)				0.332
Curative	528 (77.1)	392 (75.8)	136 (81.0)	
Palliative	111 (16.2)	87 (16.8)	24 (14.3)	
Curative, discontinued	46 (6.7)	38 (7.4)	8 (4.8)	
Treatment received—no. (%)				0.046 ^1^
Palliative/BSC	49 (7.2)	41 (8.0)	8 (4.8)	
Pall. R(C)T	46 (6.8)	36 (7.0)	10 (6.1)	
Surgery	258 (38.1)	179 (35.0)	79 (47.9)	
Surgery + adj. R(C)T	101 (14.9)	78 (15.2)	23 (13.9)	
Def. R(C)T	212 (31.3)	171 (33.4)	41 (24.8)	
Pall. CT	11 (1.6)	7 (1.4)	4 (2.4)	
Treatment recommendation—no. (%)				0.002 ^1^
Palliative/BSC	16 (2.5)	12 (2.5)	4 (2.5)	
Pall. R(C)T	51 (7.9)	42 (8.6)	9 (5.6)	
Surgery	216 (33.3)	143 (29.2)	73 (45.6)	
Surgery + adj. R(C)T	133 (20.5)	104 (21.3)	29 (18.1)	
Def. R(C)T	225 (34.7)	184 (37.6)	41 (25.6)	
Pall. CT	8 (1.2)	4 (0.8)	4 (2.5)	
Adherence—no. (%)				0.038
Non-adherent	89 (13.9)	75 (15.5)	14 (8.9)	
Adherent	551 (86.1)	408 (84.5)	143 (91.1)	

HNSCC, head and neck squamous cell carcinoma; UICC, Union for International Cancer Control; BSC, Best Supportive Care; Pall. R(C)T, palliative radio(chemo)therapy; Pall. CT, palliative chemotherapy; Adj. R(C)T, adjuvant radio(chemo)therapy; Def. R(C)T, definitive radio(chemo)therapy. ^1^ The requirements to perform a chi-square test were not fulfilled.

**Table 2:** Cross-table relating the UICC stage to the causes of death.

		UICC Stage		Total
		I–II	III–IV	
Death due to cancer	Survived	158 (53.9%)	135 (46.1%)	293 (100.0%)
	Non-cancer-associated	34 (49.3%)	35 (50.7%)	69 (100.0%)
	Cancer associated	59 (23.0%)	197 (77.0%)	256 (100.0%)
Total		251 (40.6%)	367 (59.4%)	618 (100.0%)

UICC, Union for International Cancer Control.

**Table 3:** Univariate analysis of clinicopathologic variables associated with overall survival.

Univariate Analysis										
Variable		Total			Current/Former Smokers			Non-Smokers		
		*n* = 688	Mean OS (Months/% ^1^)	*p* Value	*n* = 520	Mean OS (Months/% ^1^)	*p* Value	*n* = 168	Mean OS (Months/% ^1^)	*p* Value
Sex				0.515			0.868			0.519
	Male	493	57/39.2		390	55/36.7		103	63/50.3	
	Female	195	58/40.4		130	48/34.1		65	75/54.6	
Age at initial diagnosis of HNSCC				0.073			<0.001			0.094
	70–74 years	356	63/41.4		302	59/37.8		54	83/63.2	
	75–79 years	225	53/40.3		159	47/38.0		66	62/45.8	
	80–84 years	71	39/33.8		45	32/21.7		26	51/57.8	
	85–89 years	25	46/31.7		12	42/27.3		13	46/38.5	
	Older than 90 years	11	19/27.7		2	4/0.0		9	23/34.6	
Tobacco exposure				0.001						
	Non-smokers	168	69/52.0							
	Current/former smokers	520	54/36.0							
Alcohol abuse				<0.001			<0.001			0.004
	No ethanol consumption	317	64/49.3		209	57/46.0		108	77/56.5	
	Ethanol consumption	186	41/25.0		169	41/24.8		17	32/26.5	
Additional cancer diagnoses				0.430			0.280			0.186
	Other cancers	244	53/35.5		198	48/31.0		46	81/63.9	
	No	444	60/42.2		322	57/39.9		122	64/48.0	
Karnofsky performance status				<0.001			<0.001			<0.001
	≤70%	370	38/23.0		292	36/21.4		78	46/30.2	
	≥80%	315	79/58.4		225	77/54.7		90	86/68.1	
Charlson comorbidity index				<0.001			<0.001			0.014
	≤5	444	68/47.1		330	65/43.5		114	75/58.3	
	≥6	244	39/25.9		190	33/23.0		54	52/38.7	
Site of primary tumour				<0.001			0.004			<0.001
	Oropharynx	202	59/40.4		155	51/33.8		47	85/64.1	
	Oral cavity	240	51/37.7		173	48/35.1		67	58/46.9	
	Larynx	154	69/47.2		116	67/45.0		38	77/55.0	
	Hypopharynx	62	29/22.4		56	29/21.8		6	32/25.0	
	Paranasal sinus	24	55/50.3		16	51/52.4		8	62/46.9	
	Nasopharynx	6	19/16.7		4	27/25.0		2	5/0.0	
P16 in Oropharynx-Carcinoma				0.022			0.283			0.209
	Positive	69	72/49.2		46	52/36.1		23	106/72.4	
	Negative	66	42/33.8		59	41/31.4		7	51/64.3	
Grading				0.107			0.074			0.605
	G1	59	61/45.9		42	55/41.4		17	77/58.6	
	G2	412	61/41.1		319	58/38.5		93	68/51.7	
	G3	160	50/34.1		117	45/28.5		43	66/50.7	
T classification				<0.001			<0.001			<0.001
	T1–2	341	77/52.1		249	71/48.7		92	88/63.2	
	T3–4	347	38/27.1		271	34/24.1		76	49/38.4	
N classification				<0.001			<0.001			0.063
	Positive	347	47/30.2		271	42/26.0		76	62/45.9	
	Negative	341	65/48.9		249	62/46.3		92	73/57.1	
M classification				<0.001			<0.001			<0.001
	Positive	30	18/7.9		23	16/4.6		7	32/28.6	
	Negative	658	61/41.0		497	57/37.5		161	71/53.0	
UICC stage (8th edition)				<0.001			<0.001			<0.001
	0–II	272	77/56.7		197	71/53.1		75	98/67.0	
	III–IV	416	42/28.4		323	40/25.2		93	50/40.2	
Treatment received				<0.001			<0.001			<0.001
	Palliative/BSC	49	10/2.8		41	10/3.8		8	9/0.0	
	Pall. R(C)T	46	12/12.2		36	9/3.9		10	22/48.0	
	Surgery	258	78/59.6		179	74/55.5		79	86/72.1	
	Surgery + adj. R(C)T	101	62/37.7		78	60/34.9		23	74/50.2	
	Def. R(C)T	212	46/33.1		171	43/32.0		41	55/37.3	
	Pall. CT	11	9/18.2		7	9/0.0		4	7/25.0	
Treatment recommendation				<0.001			<0.001			<0.001
	Palliative/BSC	16	8/0.0		12	7/0.0		4	13/0.0	
	Pall. R(C)T	51	12/10.9		42	9/3.5		9	29/66.7	
	Surgery	216	82/63.1		143	78/59.4		73	89/72.9	
	Surgery + adj. R(C)T	133	62/37.7		104	60/36.2		29	68/45.8	
	Def. R(C)T	225	42/30.1		184	39/28.9		41	50/35.1	
	Pall. CT	8	15/0.0		4	12/0.0		4	17/50.0	
Adherence to treatment recommendation				<0.001			<0.001			<0.001
	Non-adherent	89	26/16.9		75	27/17.3		14	15/21.4	
	Adherent	551	66/44.2		408	62/40.3		143	76/56.6	
Recurrence				0.801			0.669			0.803
	Positive	152	53/34.4		111	49/34.2		41	61/31.4	
	Negative	536	59/41.2		409	55/36.8		127	70/57.0	
Implementation of therapy				<0.001			<0.001			<0.001
	Discontinued	44	13/7.6		41	14/8.1		3	5/0.0	
	Rejected	47	37/26.1		36	39/27.7		11	17/27.3	
	Carried out	585	64/43.1		434	60/39.4		151	73/55.1	
Intention of therapy				<0.001			<0.001			<0.001
	Curative	528	69/47.0		392	65/43.4		136	77/58.6	
	Palliative	111	14/6.1		87	11/3.0		24	16/22.1	
	Curative, discontinued	46	44/31.2		38	45/32.3		8	22/37.5	

OS, overall survival; HNSCC, head and neck squamous cell carcinoma; UICC, Union for International Cancer Control; BSC, Best Supportive Care; Pall. R(C)T, palliative radio(chemo)therapy; Pall. CT, palliative chemotherapy; Adj. R(C)T, adjuvant radio(chemo)therapy; Def. R(C)T, definitive radio(chemo)therapy. ^1^ Proportion of patients alive after a follow-up period of 60 months.

**Table 4:** Multivariate analysis of clinicopathologic variables associated with overall survival.

Multivariate Analysis					
Variable		*n* = 688	HR	95% CI	*p* Value
Age at initial diagnosis of HNSCC			1.303	1.055–1.609	0.014
	≤75	410			
	≥76	278			
Tobacco exposure			1.475	1.134–1.919	0.004
	Non-smokers	168			
	Current/former smokers	520			
Charlson comorbidity index			1.471	1.194–1.813	<0.001
	≤5	444			
	≥6	244			
Karnofsky performance status			0.504	0.405–0.627	<0.001
	≤70%	370			
	≥80%	315			
UICC stage (8th edition)			2.177	1.745–2.716	<0.001
	0–II	272			
	III–IV	416			
Current/Former Smokers					
Variable		*n* = 520	HR	95% CI	*p* Value
Age at initial diagnosis of HNSCC			1.404	1.116–1.766	0.004
	≤75	339			
	≥76	181			
Charlson comorbidity index			1.508	1.198–1.898	<0.001
	≤5	330			
	≥6	190			
Karnofsky performance status			0.514	0.403–0.655	<0.001
	≤70%	292			
	≥80%	225			
UICC stage (8th edition)			2.031	1.593–2.589	<0.001
	0–II	197			
	III–IV	323			
Non-Smokers					
Variable		*n* = 168	HR	95% CI	*p* Value
Age at initial diagnosis of HNSCC			0.886	0.531–1.478	0.643
	≤75	71			
	≥76	97			
Charlson comorbidity index			1.341	0.805–2.232	0.260
	≤5	114			
	≥6	54			
Karnofsky performance status			0.443	0.260–0.754	0.003
	≤70%	78			
	≥80%	90			
UICC stage (8th edition)			2.974	1.729–5.117	<0.001
	0–II	75			
	III–IV	93			

HNSCC, head and neck squamous cell carcinoma; UICC, Union for International Cancer Control; HR, hazard ratio; 95%CI, 95% confidence interval.

## Data Availability

The data are presented in Table 1, Table 2, Table 3 and Table 4.
